# Sleep in women: a narrative review of hormonal influences, sex differences and health implications

**DOI:** 10.3389/frsle.2023.1271827

**Published:** 2023-12-19

**Authors:** Monica L. Andersen, Helena Hachul, Isabela Antunes Ishikura, Sergio Tufik

**Affiliations:** ^1^Departamento de Psicobiologia, Universidade Federal de São Paulo (UNIFESP), São Paulo, Brazil; ^2^Sleep Institute, Associação Fundo de Incentivo à Pesquisa, São Paulo, Brazil

**Keywords:** sleep, menopause, hormonal, pregnancy, insomnia, obstructive sleep apnea, women

## Abstract

Sleep is a fundamental biological behavior that affects various aspects of health and wellbeing. However, there are some differences in respect of sleep between men and women. Notably, there are sex differences in relation to sleep problems and the potential comorbidities, such as diabetes and cardiovascular diseases, that are associated with these problems, with some evidence suggesting that women may have a greater predisposition to sleep disturbances. This narrative review provides a comprehensive analysis of the literature in respect of sex differences in the sleep, with the main focus being on women. Basic research has investigated sex-specific distinctions in sleep architecture, sleep quality, and circadian rhythms, while clinical studies have examined sex differences in sleep disorders, such as insomnia, sleep apnea, and restless leg syndrome. This narrative review also highlights the impact of the periods of hormonal fluctuations that occur across a woman's lifespan - such as during the menstrual cycle, pregnancy, and menopause phase - and examines their effects on sleep. It also explores the influence of social and cultural factors on sleep patterns in women. Taken together, the evidence suggests that women may be more susceptible to sleep disturbance, and that gender-specific factors should be considered when evaluating sleep in clinical practice. Further research is warranted to elucidate the mechanisms that underlie this and help guide the development of sex-specific interventions to improve sleep quality and promote holistic health in women.

## 1 Introduction

Sleep patterns tend to shift with age in both men and women; however, there are significant differences in sleep patterns between male and female individuals, although it is not clear exactly when these differences first appear. Studies investigating sleep in newborns indicate that sleep is consolidated at around 3 months of age, but remains very dynamic up until 6 months of age (De Beritto, 2020); however, few studies have examined sex distinctions in newborn sleep, with some suggesting that there are no sex differences, although others have reported longer “active sleep” periods in boys and more “quiet sleep” periods in girls (Paul et al., 2003; Cubero-Rego et al., 2018).

From childhood until puberty, no major sex differences have been reported in respect of sleep (Feinberg and Campbell, 2010). It is only after menarche, or 1^st^ menstruation, that differences in female and male sleep become evident. These include an increase in the frequency range of sleep spindles in non-REM sleep in women; a significant increase in the risk of insomnia in comparison to men; and reduced subjective sleep quality in women (Pengo et al., 2018). During the reproductive period, studies report changes in the sleep quality according to the phase of the menstrual cycle (Baker and Driver, 2004; Koikawa et al., 2020). Variations in the levels of progesterone and estrogen have been associated with sleep disruption (Antunes et al., 2006), and pain during menstruation has been linked to poorer sleep quality (Araujo et al., 2011). Women with irregular menstrual cycles present a 2-fold higher risk of insomnia (Antunes et al., 2006). This evidence confirms the hypothesis that hormonal alterations can negatively impact sleep quality (Hachul et al., 2010).

Thus, it is clear that several factors play crucial roles in influencing sleep quality in women. These encompass both intrinsic and extrinsic factors. In terms of intrinsic factors, hormonal levels, pregnancy, menopause, and sleep disorders all contribute to shaping the sleep experience of women. Particularly noteworthy is the impact of hormonal fluctuations across the different stages of a woman's life, as well as within the menstrual cycle itself. These fluctuations have been robustly associated with significant alterations in sleep patterns (Pengo et al., 2018). One period of a woman's life that significantly impacts her wellbeing is the climacteric phase. The climacteric represents a pivotal transitional period, involving perimenopause, menopause, and post-menopause. Menopause is characterized by the absence of menstruation for 12 consecutive months due to the decline in ovarian hormones (estrogen and progesterone) (Takahashi and Johnson, 2015). Typically manifesting between the ages of 45 and 55 years, the process of menopause entails a complex interplay of endocrinological changes consequent to ovarian functional deterioration (Takahashi and Johnson, 2015). Linked intricately with the fluctuating and gradual decline of estrogen, menopausal symptoms emerge, including vasomotor symptoms (hot flashes and night sweats), sleep disturbances, and mood alterations (Takahashi and Johnson, 2015).

Among the spectrum of menopausal symptoms, sleep disturbances emerge as some of the most vexing, reported by 40 to 60% of menopausal women (Baker et al., 2018). These sleep dysfunctions comprise sleep onset difficulties, nocturnal awakenings, and premature morning arousals (Baker et al., 2018). Elevations in cortisol levels, stemming from heightened stress peaks, exert a pronounced influence on mood and sleep quality within the climacteric phase (Baker et al., 2018). The diminishing estrogen levels further amplify vasomotor symptoms, inducing episodes of facial and anterior thoracic heat waves accompanied by sweating during the nocturnal hours, compromising sleep quality (Baker et al., 2018). The presence of menopause-related symptoms, coupled with suboptimal sleep quality, contribute to the onset or exacerbation of other conditions, such as anxiety and depression, in addition to vasomotor and sexual symptoms (Nelson, 2008). These factors can impact autonomy, independence in daily activities, and overall quality of life for women (Minkin, 2019). Understanding sleep patterns in menopausal women, and the intricate relationship between sleep and hormones, is vital for devising coping strategies and identifying interventions that can aid in developing sex-specific interventions that can benefit symptomatic women by improving sleep quality and ameliorating the adversities associated with this phase of life.

It should be remembered that sex and gender are distinct concepts that refer to different aspects of human identity. Sex refers to the biological and physical characteristics typically associated with being man or woman (Bewley et al., 2021). It is determined by factors, such as reproductive organs, chromosomes, and hormones. At birth, individuals are often assigned a sex based on these physical attributes, which is commonly referred to as “assigned sex at birth” (Bewley et al., 2021). On the other hand, gender is a social and cultural construct which encompasses the roles, behaviors, and expectations that society attributes to both men and women. Gender identity, which is personal, refers to an individual's sense of being man, woman, or another gender. It may or may not align with the sex assigned at birth (Bewley et al., 2021). While sex is often seen as a binary concept (man or woman), there is biological and medical recognition of intersex variations, where individuals may possess physical attributes that do not fit typical male or female categories (Bewley et al., 2021). Understanding and respecting the differences between sex and gender is crucial for promoting inclusivity and recognizing the diversity of human experiences. In this study, we used the terms “woman” or “man” to refer to individuals based on their biologically determined sex.

This narrative review intends to explore the various factors that influence sleep patterns across different stages of a woman's life. We incorporate recent key findings on this subject and engage in a discourse regarding the criticality of investigating the hormonal changes related to sleep disorders in animal models, as well as looking at the results from a major Brazilian sleep study.

Below, we look in more detail at the hormonal dynamics discussed above and how these change over the course of a woman's life, including during pregnancy. We also consider the psychological factors than can affect women's sleep. In addition, we look at specific human and animal studies conducted by our group and describe how these have informed our understanding of sleep in women. By doing this, we hope to shed light on the current understanding of sleep disturbances in women and potential novel strategies in this area.

## 2 Hormonal dynamics and sleep patterns in different stages of women's life

### 2.1 Effects of progesterone on sleep

**Figure 1:**
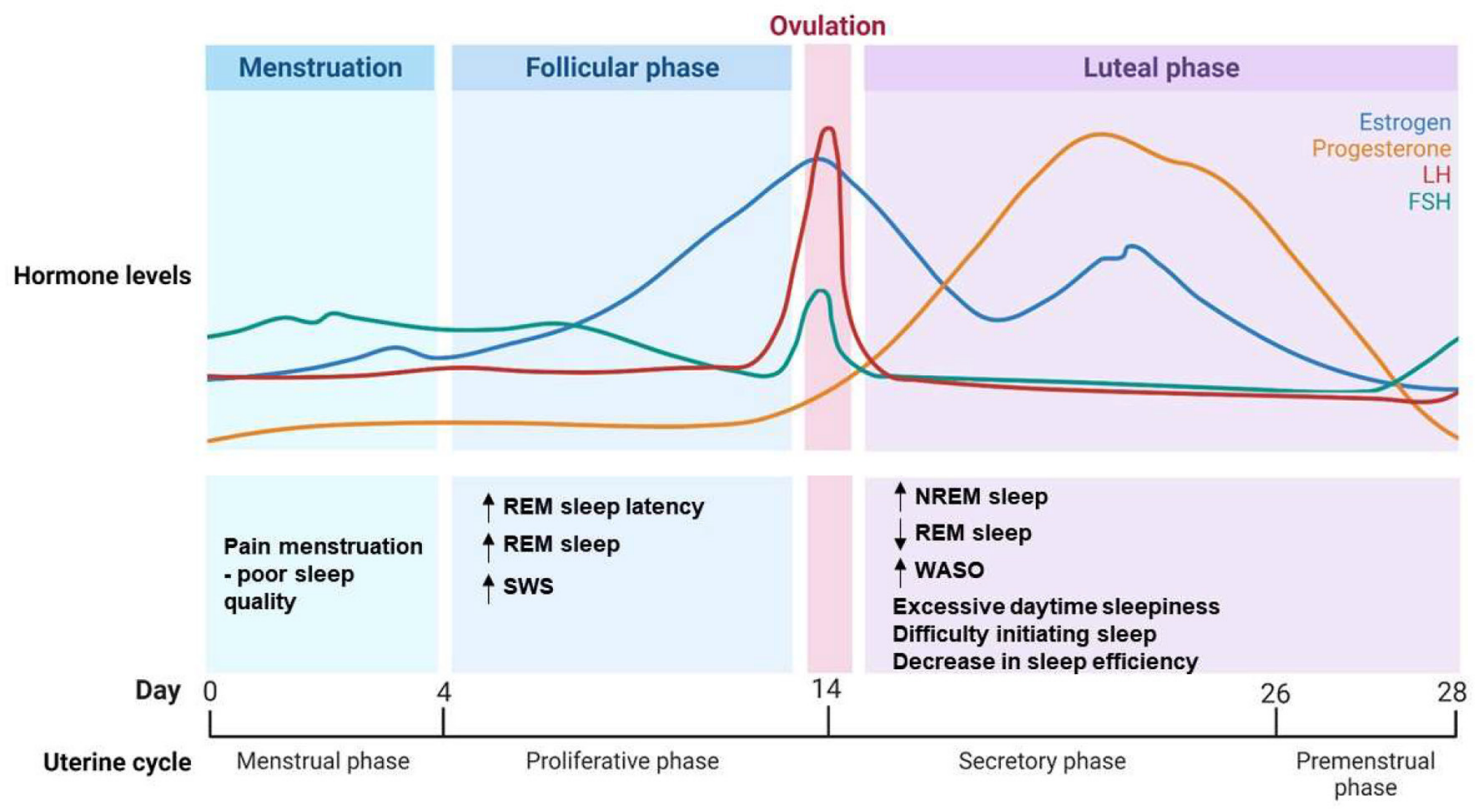
Hormonal dynamics and sleep pattern changes during the menstrual cycle. Illustration depicting the intricate interplay between hormonal dynamics and alterations in sleep patterns across the menstrual cycle. Progesterone and estrogen levels fluctuate, influencing sleep quality and sleep stages during different phases of the cycle. The graph highlights key hormonal transitions and their corresponding impacts on sleep architecture. NREM, Non-Rapid Eye Movement; REM, Rapid Eye Movement; SWS, Slow Wave Sleep; WASO, Wake After Sleep Onset; LH, Luteinizing Hormone; FSH, Follicle-Stimulating Hormone.

Progesterone is one of the most important hormones in relation to sleep regulation in women (Tobias et al., 2021). Progesterone levels peak in the luteal phase and then decrease during the menstrual period. It has been shown to enhance sleep duration and sleep quality by promoting slow wave sleep (SWS) (Figure 1) (Tobias et al., 2021). Studies have shown that progesterone has hypnotic and sedative properties in female animals and in women (Deurveilher et al., 2009; Collins et al., 2012). However, a rapid increase in progesterone during the early luteal phase leads to increased wake time after sleep onset in women (Figure 1) (Pengo et al., 2018). This intricate hormonal interplay which can affect sleep quality, intertwines with the effects of premenstrual syndrome (PMS). PMS is a complex medical condition that involves various physical, emotional, and psychological symptoms that are experienced by some women in the days leading up to menstruation during the luteal phase of their menstrual cycle (Nicolau et al., 2018). These symptoms include mood swings, irritability, breast tenderness, abdominal discomfort, and fatigue (Nicolau et al., 2018). While the exact cause is still a subject of study, hormonal fluctuations, particularly in respect of estradiol and progesterone levels, are believed to be central to the development of PMS symptoms. These symptoms can significantly affect the overall wellbeing and daily functioning of those affected (Nicolau et al., 2018). The decline in estradiol and progesterone levels toward the end of the luteal phase is associated with an increased occurrence of headaches and other PMS symptoms, which are known to disrupt sleep in women (Baker and Lee, 2018; Nicolau et al., 2018; Pengo et al., 2018; Brown and Gervais, 2020). Our previous findings revealed that women with PMS presented poorer sleep quality, a higher self-perception of unrefreshing sleep and more subthreshold insomnia symptoms compared to women without PMS. Increased total sleep time in participants with PMS was also observed in polysomnographic examinations (Nicolau et al., 2018).

### 2.2 Effects of estrogen on sleep

Estradiol, a form of estrogen and an ovarian hormone that peaks during the follicular phase, also affects sleep. It has been suggested that estradiol plays a role in the sleep-wake cycle by facilitating wakefulness, and that it has a protective effect against sleep-disordered breathing (Pengo et al., 2018; Brown and Gervais, 2020). High levels of both progesterone and estrogen, particularly during the early luteal phase, have been associated with increased wakefulness and sleep impairment (Figure 1) (Pengo et al., 2018). Progesterone and estrogen can modulate neurochemical transmission, increasing wake-promoting substances and decreasing sleep-promoting neurotransmitters (Dorsey et al., 2021). Moreover, these hormones affect glial morphology and even the activity of clock genes, altering sleep related factors such as nesting behavior, locomotor activity and circadian rhythms (Dorsey et al., 2021). Estrogen modulates sleep by promoting wakefulness, reducing REM and non-REM sleep duration, and influencing adaptation to stress and the consolidation of memory and fear extinction learning (Cabrera et al., 2020; Dorsey et al., 2021). This is to be expected, given that estrogen and progesterone receptors are found in several sleep/wake regulatory anatomic regions (nuclei), including the basal forebrain, the hypothalamus, the dorsal raphe nucleus, and the locus coeruleus (Dorsey et al., 2021).

### 2.3 Menstrual cycle and sleep

During the luteal phase, when women experience moderate estrogen and high progesterone levels, most sleep complaints are related to excessive daytime sleepiness, difficulty initiating sleep and a decrease in sleep efficiency (Figure 1) (Baker et al., 2007). The luteal phase is characterized by selective increases in the percentage of non-REM sleep, reduced REM sleep, and increased wake time after sleep onset (Figure 1) (Baker et al., 2007). The extent of the luteal phase reflecting absolute or fluctuating levels of ovarian hormones warrants further investigation. In contrast, during the follicular phase, in which ovarian hormones are generally low, although estrogen rises toward the end, women frequently report less sleep complaints and higher subjective sleep quality (Baker and Lee, 2018). Objective polysomnography studies have indicated increased REM sleep latency during this phase, and increases in SWS and REM sleep, suggesting better overall sleep quality (Baker et al., 2007; Pengo et al., 2018; Brown and Gervais, 2020). One of the most limitations found when investigating sleep in women is the difficult to obtain PSG data and hormonal levels of the whole menstrual cycle; and the application of questionnaires becomes the first choice, even being a subjective tool with self-report information. Sleep studies often report poor sleep quality and insomnia symptoms in women (Hachul et al., 2010; Baker and Lee, 2018; Kang et al., 2019). Gynecological dysfunctions, such as endometriosis, dysmenorrhea, PCOS, abnormal uterine bleeding, are very prevalent during reproductive phase of women and may unbalance hormonal release in women affecting daily routine and sleep (Kennedy et al., 2022).

### 2.4 Menopause and sleep

It is not surprising that the perimenopause and menopause are associated with alterations in the sleep quality and in sleep-wake cycles. In fact, during perimenopause, which is characterized by a gradual decline in ovarian hormone levels and increased fluctuation in hormone levels, up to 40% of women report poor sleep, with increased insomnia symptoms, restless legs syndrome (RLS) and OSA, among other complaints (Ciano et al., 2017; Pengo et al., 2018).

**Figure 2:**
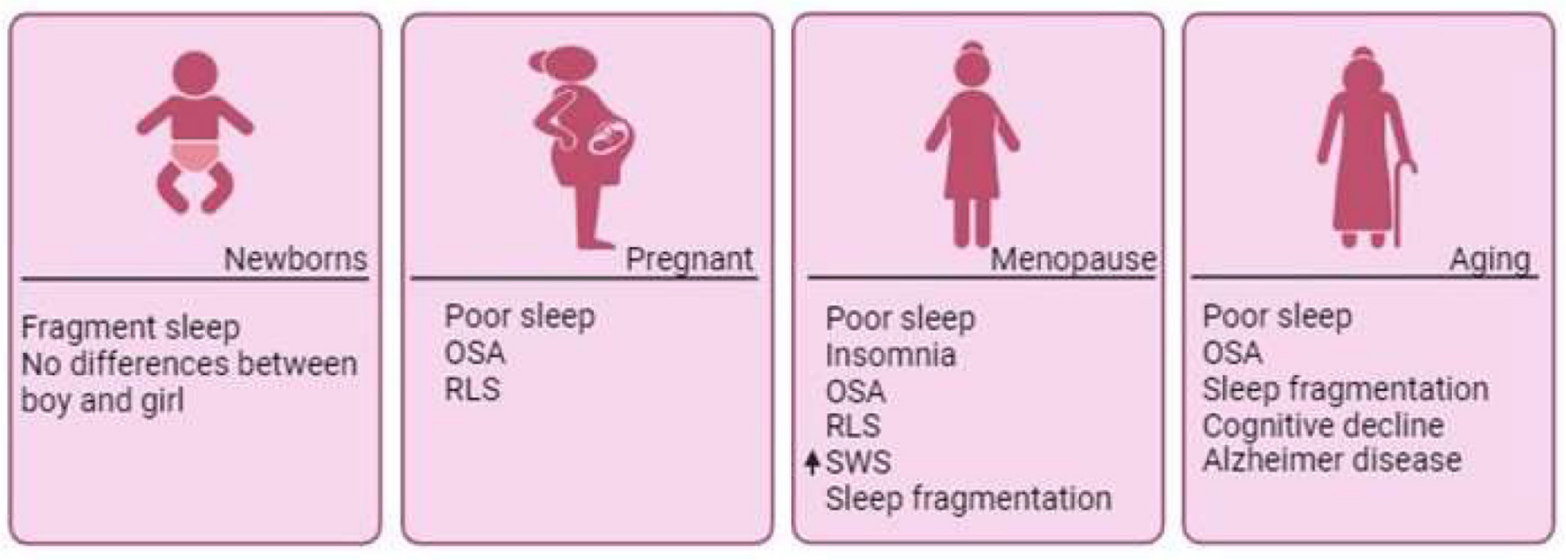
Sleep disorders across phases of a woman's life. Throughout a woman's lifespan, she undergoes numerous changes that directly impact sleep quality. Although minimal differences have been documented during the earliest days of life, significant shifts emerge with the onset of hormonal fluctuations. Pregnancy is often associated with poor sleep quality and sleep disorders. Across the entire span of menopause, the marked decline in estradiol contributes to the emergence of sleep disorders, such as OSA, insomnia, and RLS, which persist post-menopause. In the aging process, compromised sleep quality becomes associated with cognitive decline and neurodegenerative diseases like Alzheimer's disease. OSA, Obstructive Sleep Apnea; RLS, Restless Legs Syndrome; SWS, Slow Wave Sleep.

Even with the stabilization of hormone levels that can occur after the menopause, the prevalence of sleep complaints in postmenopausal women increases dramatically, with up to 60% of women reporting poor sleep quality (NIH State-of-the-science conference statement on management of menopause-related symptoms, 2005). Sleep problems, such as insomnia, RLS and OSA continue to be prevalent in this population (NIH State-of-the-science conference statement on management of menopause-related symptoms, 2005). Menopause has been associated with alterations in sleep architecture, including worse total sleep time and sleep efficiency, in addition to fragmented sleep; and increased SWS (Figure 2) (Pengo et al., 2018). OSA increases after menopause, and this is related to an increase in the apnea-hypopnea index (AHI) and a decrease in the oxygen saturation (SaO_2_) compared to perimenopausal women (Naufel et al., 2018). OSA is especially prevalent in late post-menopause. There is also a correlation between waist circumference (WC), menopausal stage, and OSA severity (Naufel et al., 2018). Each centimeter increase in WC increases the risk of OSA by 5% in post-menopause (Polesel et al., 2015). The fact that hormone therapy has been confirmed to improve sleep-related symptoms further emphasizes the relationship between hormone variations during peri- and post-menopause and sleep complaints (Hachul et al., 2015; Brown and Gervais, 2020). Hormone therapy helps improving hot flashes, decreasing night awakenings which leads to better latency and quality of sleep (Caretto et al., 2019).

It is important to note that not all changes in sleep after menopause are necessarily related to hormonal changes. Aging itself affects sleep quality in women independently from the hormonal transformations that occur in menopause. Studies have reported that older women, in addition to the overall reduction of the total sleep time and sleep efficiency observed with aging in both women and men, presented a higher number of awakenings, as well as a disrupted circadian rhythm, which is linked to decreased melatonin production (Ohayon et al., 2004; Kotlarczyk et al., 2012; Chen et al., 2014; Gursoy et al., 2015). However, results indicated that menopause itself provides a modest, but important, influence on objective sleep patterns, independently of age, particularly on the AHI and SaO_2_ parameters (Cabrera et al., 2020). Notably, changes in sleep patterns in aging women have been associated with cognitive decline and an increased risk of the Alzheimer's disease (Figure 2) (Pengo et al., 2018).

### 2.5 Sleep challenges during pregnancy: hormonal, physiological, and social factors

Several mechanical and physiological factors can influence sleep during pregnancy, including increased levels of progesterone and prolactin, diaphragmatic elevation, fetal movements, bladder distension, temperature fluctuations, gastrointestinal symptoms, weight gain and decreased respiratory function (Christian et al., 2019). Nocturia and muscle discomfort can disturb sleep throughout the entire pregnancy (Christian et al., 2019). Starting from the 2^nd^ trimester, uterine contractions and fetal movement can interfere in sleep quality, as can the increased prevalence of rhinitis and nasal congestion (Christian et al., 2019). In the 3^rd^ trimester, sleeping position, leg cramps, heartburn, and orthopnea, begin to affect sleep quality (Christian et al., 2019). Studies have reported that a rate of 46% of pregnant women experience poor sleep, which worsens during the 3^rd^ trimester (Pengo et al., 2018; Christian et al., 2019). In addition, during pregnancy there is an increased risk of RLS, which can have a negative effect on sleep (Pengo et al., 2018). When compared to nulliparous women (who have not given birth), multiparous women (who have given birth multiple times) report worse overall sleep quality, reduced sleep duration, poorer sleep efficiency and more frequent occurrence of sleep disturbances (e.g., night time and early morning awakenings) in the 3^rd^ trimester of pregnancy (Christian et al., 2019).

The importance of sleep for physiological wellbeing becomes clear during pregnancy, given that several adverse pregnancy studies have been associated with poor sleep quality. It has been shown that there is a connection among sleep-disordered breathing, gestational diabetes and hypertension, while insomnia, OSA and poor sleep have been linked to preterm delivery (Facco et al., 2017; Wilson et al., 2018). Placental hypoxia and inflammation have been proposed as the main mechanisms underlying the relationship between poor sleep and adverse outcomes in pregnancy (Ravishankar et al., 2015).

The hormonal fluctuations experienced during pregnancy, including increased levels of estrogen and progesterone, may affect sleep patterns. However, the influence of sleep disturbances during pregnancy goes beyond hormonal changes. Social factors, such as stress, lifestyle adjustments, and the quality of support systems, also impact sleep quality in expectant mothers (Grussu et al., 2005). The demands and responsibilities associated with pregnancy, coupled with societal expectations and cultural norms, can contribute to sleep disruption and difficulties in maintaining a regular sleep schedule. Moreover, sex differences play a role in determining the prevalence and manifestation of sleep disorders in pregnant women. Certain sleep disorders, including sleep apnea, RLS, and insomnia, may be more commonly observed in women during pregnancy compared to men (Pengo et al., 2018). Hormonal fluctuations, changes in the body composition, and anatomical differences may contribute to these sex-specific sleep disturbances. It is crucial to recognize and address these factors in order to promote better sleep health in expectant mothers. By providing appropriate support, education, and tailored interventions, healthcare professionals can help mitigate the negative impact caused by sleep disorders on both maternal and fetal wellbeing.

## 3 Cultural and ethnic diversity: shaping women's sleep patterns

The realm of sleep is not solely dictated by biological factors; cultural and ethnic influences play a substantial role in shaping individuals' sleep patterns and preferences, particularly among women (Yip and Cheon, 2020). Delving into the intricate tapestry of diverse cultural backgrounds reveals a spectrum of attitudes and practices surrounding sleep, with a particular focus on napping habits.

In many cultures, sleep is not merely a biological necessity but is deeply intertwined with societal norms, rituals, and familial structures (Yip and Cheon, 2020). Cultural attitudes toward sleep duration, timing, and even the perceived importance of napping can significantly vary. For women, these cultural nuances can impact their sleep patterns in various ways. Certain cultures prioritize a siesta or afternoon nap, viewing it as a rejuvenating practice essential for overall wellbeing (Valencia-Flores et al., 1998). Women from these cultural backgrounds may incorporate short naps into their daily routines, recognizing them as a culturally sanctioned and health-promoting habit (Alcántara et al., 2021). In contrast, other cultures may prioritize consolidated nighttime sleep and discourage daytime napping (Alcántara et al., 2021).

Ethnic diversity also contributes to variations in sleep patterns among women (Alcántara et al., 2021). Practices rooted in cultural traditions, such as meditation or relaxation techniques before bedtime, can influence the quality and duration of sleep. Cultural expectations related to familial roles and responsibilities may impact sleep, with women from some ethnic backgrounds experiencing unique pressures that influence their sleep habits (Alcántara et al., 2021). The impact of migration and acculturation cannot be understated. Women navigating between their cultural heritage and a new cultural context may undergo shifts in sleep patterns and preferences (Alcántara et al., 2021). Acculturation might introduce changes in lifestyle, work schedules, and exposure to artificial light, all of which can influence sleep habits (Alcántara et al., 2021). A cross-sectional study focused on the acculturative stress experienced by Korean American immigrants (KAIs) and its impact on sleep duration, examining gender differences (Park et al., 2020). Involving 343 middle-aged KAIs, the study found that, after adjusting for covariates, higher homesickness and lower civic disengagement were associated with shorter sleep duration in women. Conversely, higher isolation was linked to shorter sleep duration in men (Park et al., 2020). The results underscore gender-specific associations between acculturative stress domains and sleep duration, emphasizing the importance of considering these differences in developing interventions to enhance sleep health among KAIs (Park et al., 2020).

Another study focused on sleep disparities in a multi-ethnic sample of women participating in a culturally tailored wellness coaching program, emphasizing the modifiable contribution of sleep duration to increased cardiometabolic risks in communities of color (Simonsen et al., 2023). Among 485 women, 41% reported short sleep duration (<7 h). Adjusted models revealed higher odds of short sleep duration for Blacks/African Americans and Native Hawaiians/Pacific Islanders compared to Hispanics/Latinas (Simonsen et al., 2023). Depression symptoms and stress management scores were associated with short sleep. Only 10.7% expressed interest in sleep improvement, with African Immigrants/Refugees and African Americans showing less interest. Community leaders and health workers reported a lack of awareness regarding sleep's health role and discussed challenges to obtaining adequate sleep. The study highlights a gap between the high prevalence of short sleep and low interest in improvement, emphasizing the need for culturally informed interventions in diverse women (Simonsen et al., 2023).

Understanding the cultural and ethnic dimensions of sleep patterns in women involves recognizing the diversity within diverse populations. As healthcare professionals and researchers, seek to address sleep-related concerns, acknowledging and respecting these cultural and ethnic influences is essential. Tailoring sleep interventions to align with diverse cultural norms ensures a more holistic and effective approach, promoting women's overall wellbeing across different cultural landscapes.

## 4 Gender disparities in sleep: impact, influences, and health implications

Several psychological factors, directly or indirectly associated with gender, markedly impair women's sleep, including psychiatric conditions, lifestyle, stress and specific periods, such as post-pregnancy. It is known that gender-based norms regarding sleep are closely linked to social and cultural patterns that lead to unequal repercussions on women and men. For example, a study reported that experienced mothers presented more episodes of fragmented sleep and perceived worse sleep quality compared to 1^st^ time mothers (Kenny et al., 2021). Pregnancy can lead to disturbed mood, and this effect has been found to be stronger in unplanned pregnancies (Grussu et al., 2005). Following birth, postpartum blues (Grussu et al., 2005), commonly known as baby blues commonly known as “baby blues,” is a transient mood disorder that occurs in up to 85% of new mothers at 3 to 4 days post-delivery, usually dissipating within a week. A lower percentage of women experience a major perinatal depressive disorder during the pregnancy period (up to 20%) or the postpartum stage (ranging from 12 to 16%) (Mehta et al., 2015). Baby blues are associated with sleep disturbance and higher sleep complaints (Mehta et al., 2015).

Childcare and household chores are other factors that can disproportionately impact women's sleep. Although studies have indicated that there has been an increase in the amount of time that men dedicate to household chores (Mehta et al., 2015; Guppy et al., 2019), women continue to shoulder most of these responsibilities, impacting their sleep pattern. Although women's share of childcare has shrunk because of greater paternal involvement, the actual amount of time women spend on childcare has been increasing (Guppy et al., 2019). Given the complexity of the norms surrounding childcare, it could affect sleep in multifactorial ways that are not the same for all individuals. Other societal factors, such as socioeconomic pressure, society's beauty standards, stress and the “double shift” often experienced by women in respect of work and home, also affect women, and have been shown to be directly associated with impaired or insufficient sleep among women in modern society (Albuquerque et al., 2014a,b).

Given the fact that sleep loss, sleep impairment and sleep disorders are prevalent among women, understanding the ramifications of this is extremely important. Stress is one of the main factors that affects the sleep quality in women (Kloss et al., 2015). The interaction between sleep quality and stress is bidirectional. Stress responses trigger the activation of the hypothalamus-pituitary-adrenal axis and, thus, increases production of cortisol (Meerlo et al., 2002; Lateef and Akintubosun, 2020). Increased cortisol levels alter sleep quality, impairing sleep onset and consolidation (Meerlo et al., 2002). In contrast, poor sleep is one of the most commons and best studied side effects of stress, completing this cycle and creating a feedback loop that further aggravates both stress and sleep (Andersen et al., 2011; Lateef and Akintubosun, 2020).

Many of the consequences and causes of poor sleep are related to other conditions. Stress, for example, is not only linked with poor sleep, but also with mood conditions and pain, both of which are also influenced by, and can cause, sleep impairment (Catalá et al., 2023). Evidence suggests that the hormone variations that occur during a woman's menstrual cycle not only affect sleep but also mediate pain and mood disorders (Choy, 2015; Catalá et al., 2023), further emphasizing how the many components underlying sleep impairment in women are interconnected and together contribute to sleep loss (Choy, 2015; Catalá et al., 2023). Poor sleep not only affects the stress response system, but also nearly all of the systems of our body, especially the cardiovascular, endocrine and immune systems (Zager et al., 2007; Martins et al., 2008; Tenório et al., 2013). Individuals with sleep complaints or bad sleep habits are more prone to develop insulin resistance, type II diabetes, dyslipidemia, systemic inflammation, as well as suffer from cardiovascular outcomes, such as stroke and myocardial infarction (Martins et al., 2008; Naufel et al., 2018; Andersen et al., 2021). Infections can be more frequent, and even variations in the intestinal microbiome can be observed (Farré et al., 2018). While substantial insights have been gleaned from clinical investigations, it remains imperative that a concerted effort is directed toward further clinical, basic, and pre-clinical studies to unravel the intricacies of sleep-related sex differences and their implications for health. Below we describe a major population-based sleep study carried out by our group.

## 5 Sleep disorders in São Paulo: insights from the EPISONO study and their implications for women's health

We have been conducting an ongoing research project (EPISONO) at the Sleep Institute in São Paulo that investigates the prevalence of sleep disorders in the population of the city every 10 years (Pires et al., 2007; Santos-Silva et al., 2009). This project was designed to be representative of the population of the city of São Paulo and included over 1,300 participants aged from 20 to 80 years. All participants took part in a full-night polysomnography study, in addition to completing a number of questionnaires and undergoing a variety of tests (Santos-Silva et al., 2009). The results of the last EPISONO study in 2007 indicated that 48.6% of women and 42% of men met the criteria for the diagnosis of insomnia (Bittencourt et al., 2009). Importantly, 39% of the woman volunteers presented excessive daytime sleepiness (Bittencourt et al., 2009).

In agreement with data in the literature in respect of the influence of the menstrual cycle and ovarian hormones on sleep, our findings indicated that PMS was associated with poorer sleep quality, a higher self-perception of unrefreshing sleep and subthreshold insomnia. In our sample, post-menopausal women spent more time in SWS, and showed higher AHI and lower SaO_2_ compared with the perimenopausal women (Hachul et al., 2015; Lucena et al., 2020).

According to our studies, insomnia was the most prevalent sleep disorder among women, affecting nearly half of the women in the sample (Lucena et al., 2020). Obstructive sleep apnea was the 2^nd^ most prevalent sleep disorder, being found in about a quarter of the women in our sample (Tufik et al., 2022). Both conditions have similar next-day symptomatologies, including excessive daytime sleepiness, fragmented and non-restorative sleep, and changes in circadian rhythm. Insomnia and OSA in women share a number of comorbidities, including hormonal imbalances, mood disorders and chronic pain (Frange et al., 2017). Regarding the latter, sleep and pain exhibit a bidirectional relationship that affects both men and women. Common pain states in women, such as fibromyalgia, arthritis and migraine worsen sleep consolidation and directly impair sleep, while sleep impairment increases pain sensitivity, completing and propagating this vicious cycle (Frange et al., 2017, 2018).

In conclusion, the findings from the EPISONO study provide valuable insights into the prevalence of sleep disorders among the population of São Paulo. The study's comprehensive approach, which involved polysomnography, questionnaires, and various tests, shed light on the multifaceted nature of sleep disturbances in the population, and allowed us to examine specifically how women were affected. The high prevalence of insomnia and OSA among women highlights the urgent need for tailored interventions and greater awareness regarding sleep health in this demographic. The interplay between hormonal imbalances, mood disorders, and chronic pain underscores the complex nature of sleep disorders in women. As we continue to unravel the intricate dynamics of sleep disorders, these findings show the need for a comprehensive approach to women's health that takes into account not only their reproductive cycle but also the broader impact of sleep on overall wellbeing.

## 6 Unveiling sleep mechanisms and hormonal interplay: insights from animal models

Animal models are useful for the exploration of the mechanisms underlying the behavioral and physiological switches related to sleep. For example, rodent studies have proved that the relationship between changes in ovarian hormones and sleep impairment is bidirectional. They have shown that several pathways affected by sleep disturbances interfere with hormonal production, and that estradiol and progesterone levels modulate sleep (Van Cauter et al., 2008; Leproult and Van Cauter, 2010). Animal models have corroborated human studies, demonstrating that increased levels of both estradiol and progesterone are correlated with decreased sleep duration and increased wakefulness (Andersen et al., 2007). These studies have also described the different mechanisms that mediate changes in sleep and wakefulness reported in respect of ovarian hormonal level fluctuations (Dorsey et al., 2021). Moreover, animal studies enable research into the specific mechanisms of sleep alterations witnessed in females, and allow us to better understand the importance of specific sleep stages in respect of behavioral or physiological phenomena. The multiple platform method has been used in animal models to investigate the effects of selective REM (or paradoxical sleep) deprivation.

Our group has conducted sleep deprivation studies with female animals in our laboratory (Tufik et al., 2009), finding that that selective REM sleep deprivation in female rats during diestrus, which corresponds to the human late secretory stage of the reproductive cycle, led to a disrupted estrous cycle in the recovery period, indicated by a constant diestrus state during the 1^st^ week, with up to 10 days of permanent diestrus (Antunes et al., 2006), thereby reducing the total number of days of estrus in the period of recovery, and delaying the occurrence of the proestrus phase. While the proestrus is the preparatory stage for an animal coming into heat, and corresponds to the follicular stage in humans, the estrus is the phase that follows that and is the “ovulatory” period during which the animal is on heat. In a translational approach, these data imply that REM sleep deprivation alters the female reproductive cycle, and may delay and decrease the number of fertile days in females (Antunes et al., 2006). On the other hand, when female rats were submitted to REM sleep deprivation during proestrus, which, again, corresponds to the human follicular stage, we detected an increase in the number of days in estrus phase during recovery (Antunes et al., 2006). When we explored the hormonal profile connected with these changes, we found that REM sleep deprivation resulted in an increase in progesterone and corticosterone in female rats during diestrus, as well as decreasing estradiol and estrone. The stress induced by sleep deprivation is noticed by the increase in corticosterone blood levels due to activation of the hypothalamic-pituitary-adrenal axis (Nollet et al., 2020), this latter is also involved in sexual hormone release by gonadal pathway. Stress may play a significant role in the REM sleep deprivation-induced disruption of the estrous cycle. These results indicated that the diestrus is the phase of the estrous cycle which is particularly affected by stress conditions, including sleep deprivation. Of note, each phase of the estrous cycle can present a variable susceptibility to stress, with the diestrus and proestrus being the 2 phases that are uniquely modulated by REM sleep deprivation during the recovery period (Antunes et al., 2006).

In a separate study, we investigated the impact of REM sleep deprivation on circulating lipoproteins in males, and in intact and ovariectomized female rats. We found that REM sleep deprivation significantly reduced the cholesterol levels in the intact female animals compared to the ovariectomized females and male rats. REM sleep deprivation resulted in decreased triglyceride levels in all groups, except for diestrus female rats, and in increased levels of HDL in male rats relative to their respective controls (Antunes et al., 2007). REM sleep deprivation led to an increase in LDL levels in male and ovariectomized female rats, while intact females did not experience such an increase. The increase in LDL induced by REM sleep deprivation was even higher in ovariectomized females than in males. This result was completely prevented by sexual hormones in intact females, suggesting that the female hormonal cycle can significantly interact with the lipid profile related to cardiovascular risks. The similarities seen in the blood parameters between ovariectomized females and males may be result of the suppression of ovarian hormone release that occurred after ovariectomy (Antunes et al., 2007).

Sex differences can also be noted in respect of the influence of total sleep deprivation on emotional memory retrieval in mice. Both males and females presented impaired memory retrieval after total sleep deprivation in fear conditioning and passive avoidance tasks. However, there were significant sex differences when using the plus-maze discriminative avoidance test, in which the memory impairing fallout of sleep deprivation was greater in females than in males (Andersen et al., 2021). Beyond the hormonal differences between sexes, males have demonstrated increased signal-activation in memory pathways during consolidation (Gresack et al., 2009), while females' brain activates differentially amygdala and hippocampus during retrieval (Keiser et al., 2017). Alterations in molecular mechanisms and brain regions in females and males have been reported by studies focusing on memory (Tronson, 2018). All these pathways are involved in sleep regulation which would entail different memory responses to sleep deprivation between sexes. Together, these outcomes highlight the negative effect of total sleep deprivation on emotional memory retrieval, and indicate that this can be more pronounced in female mice depending on the memory task used (Fernandes-Santos et al., 2012).

We additionally considered sex differences on sleep rebound architecture after REM sleep deprivation. After a 5 day baseline sleep recording, both male and female rats in different phases of the estrus cycle were submitted to 96 h of REM sleep deprivation, and sleep rebound was then evaluated for 5 days, or one estrus cycle (Andersen et al., 2008). Our findings indicated that after REM sleep deprivation, the sleep efficiency values of all groups returned to baseline on the 2^nd^ day, except for the females that underwent sleep deprivation during the proestrus phase. A study comparing electroencephalographic parameters between males and females rats observed that proestrus phase was characterized by a decrease in non-REM and REM sleep and more time spent awake than males and other phases of the estrous cycle (Swift et al., 2020). The increased in arousal centers during proestrus may be associated to reproduction period, as proestrus is a receptive phase for female rats. The percentage of SWS remained mostly unchanged after sleep deprivation, except for the females submitted to sleep deprivation during the proestrus phase. Although all groups experienced a significant increase in REM sleep during the 1^st^ day of the rebound dark period following REM sleep deprivation, there was a distinct difference in the maintenance of this increase. Specifically, the proestrus, estrus, and diestrus-anestrus groups, in contrast to the diestrus group with regular cycles and males, continued to exhibit elevated REM sleep until the 2^nd^ day of the rebound dark period. It has been reported that male mice present lower NREM sleep rebound after sleep deprivation as well as male rats, when compared to females (Paul et al., 2006; Kostin et al., 2020). The absence of alteration in non-REM sleep amount in rebound period was observed in aging rats, irrespective of sex (Kostin et al., 2020). Regarding REM sleep amount, studies have shown contradictory results between sexes (Franken et al., 2006; Paul et al., 2006; Ehlen et al., 2013). In our study, Wistar anestrus females, as the females in proestrus, were more susceptible to REM sleep deprivation, because both manifested shifts in their sleep patterns that increased the time required to the return baseline values (Andersen et al., 2008). These findings point out the role of female hormones in sleep regulation, especially after sleep deprivation.

As mentioned previously, sleep can impact chronic pain, and chronic pain can, in turn, affect sleep patterns. These bidirectional interactions may have unique effects, and hormonal alterations appear to be involved. Thus, we investigated the long-term effects of the chronic articular pain on sleep in experimental model of osteoarthritis in both female and male rats. We recorded sleep patterns during baseline and after osteoarthritis induction, with sleep being monitored at days 1, 10, 15, 20, and 28 after induction of osteoarthritis by a iodoacetate injection in the knee joint (Silva et al., 2011). The result indicated that the overall sleep architecture was altered in both genders. Osteoarthritis produced a fragmented sleep pattern, with decreased sleep efficiency, SWS, and REM sleep and fewer REM sleep bouts regardless of sex and the estrous phase during the light, or inactive, period. However, after osteoarthritis induction, males exhibited lower sleep efficiency and reduced SWS in the dark period in contrast to females (Silva et al., 2011). Additionally, osteoarthritis affected the hormone levels of the male rats, as the testosterone rates were reduced when compared to the control and sham groups. In females, in contrast, progesterone and estradiol levels remained constant throughout the investigation. Our results revealed that the chronic model of osteoarthritis influenced sleep characteristics in both sexes. However, hormonal factors appear to contribute to the observed sex differences in this model, as male rats exhibited greater sensitivity to changes (Silva et al., 2011).

## 7 Future perspectives

The question of whether women sleep better than man may seem straightforward at 1^st^ glance, but as we dive deeper into the complexities of sleep, the answer becomes much more nuanced. There are numerous factors that can impact a woman's sleep quality, including the phase and hormone levels associated with her reproductive cycle, lifestyle factors that can directly and indirectly shape sleep patterns, and comorbidities that may affect women differently than men. These factors, and many others, can greatly influence a woman's ability to get a good night's rest, highlighting the need for further research in this area. While we may not have a definitive answer to this question yet, it is clear that there is much more to the story than meets the eye.

Given all the evidence of sex differences in sleep, precision medicine should consider sex as a factor when it comes to sleep medicine. The most marked example would be sex-specific prescribing guidelines, such as those that are already in place with zolpidem, with the FDA determining in 2018 that the zolpidem dose for female individuals should be reduced from 10 to 5 mg due to the different pharmacokinetics in men and women that can lead to higher next-morning serum levels of this substance in women 60+ years old (Greenblatt et al., 2019).

One topic of investigation that needs further attention is the conflicting findings between complaints of subjective sleep and the objectively sleep measured in women. During our epidemiological sleep studies, we have observed a higher prevalence of subjective symptoms of insomnia in women subjects compared to objective symptoms. Understanding these differences may provide us with tools to help women sleep better and feel like they have effectively slept better.

While a lot of progress has been made in the understanding of sex differences associated with sleep patterns, many knowledge gaps exist in this field, with the main reason being that women have historically been underrepresented in scientific studies. The specific mechanisms underlying how the sex steroids guide sleep circuitry remain unknown, but the use of animal models and a translational approach can help us deepen our understanding of these mechanisms.

## 8 Conclusions

It is evident that there is a profound interplay of biological and environmental factors that influence women's sleep patterns across various life stages. While sleep patterns undergo age-related changes in both men and women, distinctive sex-based differences become apparent following menarche, significantly impacting sleep frequency and quality. Hormonal fluctuations, pregnancy, and menopause assume pivotal roles in disrupting sleep quality, with menopause-related symptoms, especially sleep disturbances, affecting a significant proportion of women. These sleep disruptions, characterized by challenges in sleep onset, nocturnal awakenings, and early morning arousals, are further exacerbated by elevated cortisol levels and vasomotor symptoms, ultimately influencing the overall wellbeing and quality of life for women. A comprehensive understanding of these sleep patterns and their intricate hormonal underpinnings is imperative for the development of gender-specific interventions aimed at enhancing sleep quality and alleviating the challenges associated with the female experience.

As mentioned above, social and cultural aspects play a significant role in women's sleep patterns. While studies have reported that women may fall asleep faster than men, which could reflect better sleep quality, this may simply suggest that women have a greater sleep need and/or are more tired on average. The gender-related aspects on sleep behavior may be associated with social and cultural factors which have different, and often unequal, implications for women. Given the complexity of these factors, sleep may be affected through multifaceted routes that can vary for each individual. Caring for others, dealing with household tasks, work and the expectations of others are all factors that are known to negatively impact women more than men, and thus affect women's sleep patterns and sleep hygiene more than they affect men. It is crucial to recognize and address these gender-specific influences to promote better sleep health for women.

## Author contributions

MLA: Conceptualization, Funding acquisition, Supervision, Visualization, Writing–original draft, Writing–review & editing. HH: Supervision, Visualization, Writing–review & editing. ST: Conceptualization, Funding acquisition, Resources, Supervision, Visualization, Writing–review & editing. IAI: Writing–review & editing, Methodology, Conceptualization, Resources.
